# Caregivers’ Perceptions of the Impact of Palliative Care: A Qualitative Study

**DOI:** 10.7759/cureus.100828

**Published:** 2026-01-05

**Authors:** Catarina Vieira, Manuel Barbosa, Sílvia Marina

**Affiliations:** 1 Faculty of Medicine, University of Porto, Porto, PRT; 2 Life and Health Sciences Research Institute, School of Medicine, University of Minho, Braga, PRT; 3 Lidador Family Health Unit, São João Local Health Unit, Porto, PRT; 4 Maia/Valongo Home-Based Palliative Care Support Team, São João Local Health Unit, Porto, PRT

**Keywords:** caregiver burden, community-based palliative care, home-based palliative care, informal caregiving, palliative care, primary health care, qualitative research

## Abstract

Introduction: Population aging and the rising prevalence of multimorbidity have contributed to increased levels of dependency, placing informal caregivers at the forefront of home-based care delivery. Although palliative care (PC) aims to support both patients and their families, evidence regarding caregivers’ own perceptions and experiences of this role remains limited. Furthermore, the extent to which community PC teams shape caregivers’ lived experience is still insufficiently understood.

Methods: This qualitative phenomenological study involved conducting four semi-structured interviews with caregivers who were supported by a community PC team. Data were collected at home, audio-recorded, and fully transcribed. Content analysis was performed using pre-defined themes (Referral Process, Caregiving Experience, Expectations, Impact of PC on Caregiving Experience, Expectations vs. Reality, and Suggestions for Improvement). The saturation point was determined by consensus among the research team.

Results: All participants were women who took on most of the caregiving tasks, such as hygiene, feeding, managing medication, and accompanying patients to medical appointments. They reported fatigue, poorer sleep quality, and emotional ambivalence regarding their role. Caregivers valued the technical and relational proximity of PC, particularly home visits, accessibility via telephone, practical guidance, and coordination with rehabilitation teams. Perceived late referrals, waiting times, and shortages of human resources were recurrent themes. Social life, work, and financial domains were particularly affected, with unmet needs relating to respite, bureaucratic barriers, and access to social support.

Discussion: Community PC increased caregivers’ sense of safety, competence, and reassurance, particularly through easily accessible, home-based support. However, broader needs, particularly in relation to personal time, social participation, and navigating social and labor rights, remain insufficiently addressed. Structural limitations in formal support services and referral delays can further exacerbate caregiver burden.

Conclusion: Home-based care relies heavily on caregivers who have very limited time and substantial responsibilities. To improve the caregiving experience and promote equitable, sustainable end-of-life care at home, it is essential to strengthen early PC involvement, improve accessibility (including structured telephone support), and integrate social and labor policies, such as providing respite options, simplifying access to benefits, and protecting caregivers’ financial security.

## Introduction

The global aging population, Portugal being no exception, has resulted in an increasing number of people living with chronic illnesses and, consequently, in states of dependency [1]. This dependency creates the need for a caregiver, a role which is often taken on by a family member. As they are responsible for the majority of support delivered in the home setting, these family members, acting as informal caregivers, serve as essential pillars of care provision [2].

In palliative care (PC), particularly within community PC, evidence shows that the presence of an informal carer is often crucial in enabling patients to receive care, and ultimately to die, at home. Without family support, many patients would be unable to fulfil this preference [3].

Existing literature on the caregiving experience reveals ambivalence about this role. On the one hand, caregivers report strain, but on the other, they express satisfaction at being able to support their loved one during this time, often describing a sense of duty fulfilled. Therefore, caring for a patient, particularly one receiving PC, is marked by intense and contrasting emotions [4]. Furthermore, caring for a patient at home, particularly in the context of a palliative illness, is highly demanding, and caregiver burden is common. This burden tends to increase with the length of time spent caring for the patients [5].

According to the “Lei de Bases dos Cuidados Paliativos,” the Portuguese Palliative Care Framework Law (2012), PC is defined as “active, coordinated, and comprehensive care provided by specialized units and teams, either in inpatient settings or at home, for patients experiencing suffering due to an incurable or severe illness in an advanced and progressive stage, and for their families…” The same law defines family as “the person or persons designated by the patient or, in the case of minors or individuals without decision-making capacity, by their legal representative, with whom the patient has a close relationship, regardless of whether a biological or legal kinship exists.” As the role of informal carer often falls to family members, and given the emphasis on continuity of care in this legislation, caregivers are also included within the remit of PC teams [6,7].

Having harmful consequences for caregivers themselves, exhaustion also poses risks to patients, increasing the likelihood of mistreatment or neglect [8]. In recognition of the importance and vulnerability of informal caregivers, Portugal took a significant step in 2019 by approving the “Estatuto do Cuidador Informal” (ECI), the Informal Caregiver Statute, in 2019. This legislation established the rights and duties of informal caregivers and care recipients for the first time, as well as a set of support measures [9]. However, since its inception, the ECI has been criticized for its shortcomings and the bureaucratic complexity of its implementation. In 2024, therefore, the legislation was revised and strengthened to expand access to the ECI and streamline its procedures [10].

The emergence of government measures aimed at protecting informal caregivers is not unique to Portugal. In order to reduce caregiver burden, European countries have increasingly adopted three main types of intervention: financial support in the form of allowances and caregiver pensions; work-care reconciliation measures such as leave arrangements and flexible schedules; and direct support services including training, counseling, and respite care. An integrated approach combining these dimensions has proven to be more effective [11].

From a scientific standpoint, attention has traditionally focused primarily on patients, with fewer studies dedicated to the experiences and needs of their caregivers, particularly in relation to how these evolve over time and in relation to the supports available. Although this trend has begun to shift over the past decade, several areas remain underexplored. Consequently, further research is needed to improve understanding of caregivers’ perspectives in the context of community PC, namely, how caregivers experience the caregiving role, how they perceive the support received (including referral and follow-up), and what unmet needs they identify [12].

Therefore, it is important to understand caregivers’ perceptions of how community PC support influences their caregiving experience, including which aspects they consider most relevant (e.g., accessibility, home-based support, guidance, and links to social resources) and how they describe the impact on their daily life. Such an understanding is essential for meaningfully guiding and evaluating the quality of PC interventions, particularly in community settings. With this in mind, we propose a qualitative phenomenological study focusing on caregivers of palliative patients who are supported by a community PC team. The study aims to explore their perceptions of the caregiving role, the care provided, and how PC influences their experience of caregiving.

## Materials and methods

Study design

This is a qualitative exploratory study, using content analysis aimed at exploring caregivers’ lived experiences, before and after the involvement of community PC, and exploring how community PC influences their caregiving role. The following specific objectives were defined: to understand caregivers’ perceptions of the referral and follow-up process in PC; to examine their views on community-based PC follow-up; to explore caregivers’ expectations of support from community PC teams, and how these expectations evolve throughout the caregiving journey; and finally, to identify themes that caregivers consider relevant to the care provided to patients.

Population

The study population consists of caregivers of patients supported by a Community Palliative Care Support Team (CPCST) within the North of Portugal, integrated into the Portuguese National Health Service.

Sample

A non-randomized convenience sample was used. Inclusion criteria include being a caregiver followed by the CPCST in August 2024, agreeing to be contacted by telephone by the principal investigator, and agreeing to receive a home visit for an interview during the same month. Participants also had to be the patient’s primary caregiver (i.e., the person who provides most of the care and/or is responsible for organizing and managing it) and agree to participate voluntarily. The exclusion criteria included caregivers who did not have a personal or family relationship with the patient and caregivers whose patients had not previously been in contact with the team.

Initially, a professional from the CPCST obtained consent to share the telephone contact details of caregivers for the purpose of study recruitment. Five caregivers were identified at this stage. The principal investigator then invited each of them to participate by telephone. Four agreed to take part, and one was unavailable.

Data collection instruments

Personal and sociodemographic data were collected using a questionnaire (see Appendix A) developed specifically for this study by the research team. The following variables were recorded: gender, age, marital status, educational level, occupation, employment status, relationship to the patient, and follow-up duration by the CPCST. The questionnaire was administered orally by the principal investigator.

Qualitative data were gathered through individual semi-structured interviews, guided by a script of open-ended questions (see Appendix B). Given the homogeneity of the sample and the specificity of the study context (caregivers supported by the same community PC team), thematic redundancy across core domains emerged early in the analysis. At this point, it was deemed unnecessary to collect any more data, as this would not generate new insights and would place an avoidable burden on participants and researchers [13]. The decision was taken by consensus within the research team.

Data collection procedures

The interviews were conducted in the caregivers’ homes and were carried out by the principal investigator. Each interview lasted an average of 45 minutes. To ensure complete capture of the information provided, all interviews were audio-recorded. The recordings were then transcribed for analysis. Audio recording and verbatim transcription support the criterion of confirmability, ensuring that the study’s findings are not influenced by the researcher’s motivations, interests, or perspectives [14]. The principal investigator’s field notes taken during the interviews were also considered.

All ethical procedures inherent to this type of study were followed. Prior to data collection, the study was submitted to and approved by the Ethics Committee of ULS São João prior to data collection (project number 60/2024).

Data analysis

The interview transcripts were transcribed in full in Word format (Microsoft Corp., Redmond, WA, US) and then imported into NVivo (Lumivero LLC, Denver, CO, US) for analysis. Content analysis was used to examine the data, following the three phases defined by Bardin [15]: pre-analysis, exploration of the material, and treatment and interpretation of results.

Analytical themes were defined, some of which were further subdivided into categories. The analytical process was primarily deductive, with themes and categories established a priori to guide the coding process, in line with the study objectives and existing literature. The coding framework was continuously reassessed in light of emerging insights from the data and modified whenever justified, including the merging of initially separate categories when this proved analytically more coherent.

Coding was conducted independently by two researchers to enhance analytic credibility and confirmability. Discrepancies were resolved through discussion within the research team. Field notes and verbatim transcripts were used to support analytical rigor and to ensure that interpretations were grounded in participants’ descriptions.

Six main themes and seven categories (spread across three of the themes) were identified. The conceptual framework of these themes and categories is presented in Table 1.

**Table 1:** Conceptual Framework of the Analysis Themes and Categories Only the themes Caregiving Experience, Expectations, and Caregiving Experience-Impact of PC were subdivided into categories. *For the themes Referral Process, Expectations vs. Reality, and Suggestions for Improvement, no categories were defined; therefore, the corresponding cells are left blank. PC: palliative care

Themes	Categories
Referral Process	*
Caregiving Experience	Care dynamics (operational aspects)
	Physical
	Psychological
	Social life
	Family
	Professional/financial
	Social responses and support
Expectations (regarding PC)	Care dynamics (operational aspects)
	Physical
	Psychological
	Social life
	Family
	Professional/financial
	Social responses and support
Caregiving Experience-Impact of PC	Care dynamics (operational aspects)
	Physical
	Psychological
	Social life
	Family
	Professional/financial
	Social responses and support
Expectations vs. Reality	*
Suggestions for Improvement	*

Analytical themes

The Referral Process theme encompassed caregivers’ descriptions of how they came to be followed by the CPCST. This included information on whether the referral was made by the hospital or by the family physician. This theme was not subdivided into categories.

Caregiving Experience included caregivers’ perceptions of “being a caregiver,” incorporating all accounts that did not specifically address the role of PC. Expectations captured the expectations of caregivers regarding PC before the CPCST follow-up had begun. In Caregiving Experience-Impact of PC, caregivers described how PC had influenced their experience of caregiving. These three themes were subdivided into seven categories.

Care Dynamics encompassed caregivers’ descriptions of the practical aspects of daily life, including the care tasks and how they were organized and distributed. The “Physical/Psychological” category included accounts of how the caregiving role affected caregivers’ physical and psychological health. This category incorporated concerns or complaints relating to the health of caregivers, as well as the emotions associated with being a caregiver. Although the initial plan was to analyze the physical and psychological dimensions as two separate categories, this division proved unnatural during the content analysis, so the two were merged into a single category.

In the “Social Life” category, we considered how caregivers spent their free time and the impact that the caregiving role had on this aspect of their lives. Although this category was closely related to the previous one, its prominence in the content analysis justified maintaining it as an independent category. Therefore, whenever emotions or references to impact, such as psychological effects, were described in the context of social life, they were coded within this category.

The Family category encompassed the experience of being a caregiver within the family dynamic. In the Professional/Financial category, we considered any changes or impacts on caregivers’ employment, income, or work routines, as well as the financial burden associated with caregiving.

The Social Responses and Support category covered descriptions of care resources available to patients that were not entirely provided or initiated by the family. These included material resources, services, or financial support, such as subsidies.

In the Expectations vs. Reality category, only content involving comparative reflection was included, as opposed to the Expectations theme, which captured descriptive anticipations. This category incorporated comparisons made by caregivers between their expectations of the support they would receive from the CPCST and their actual experience.

The Suggestions for Improvement category included caregivers’ unmet needs. While expressions of dissatisfaction emerged throughout the interviews, this category only included concrete suggestions for potential changes or measures to be implemented.

## Results

Sociodemographic characteristics of caregivers

Table 2 presents the sociodemographic profile of the caregivers.

**Table 2:** Sociodemographic Profile of Caregivers CPCST: Community Palliative Care Support Team

Identification	C.46	C.47	C.48	C.49
Age	70 years	70 years	54 years	72 years
Gender	Female	Female	Female	Female
Relationship to the patient	Wife	Daughter	Daughter	Wife
Marital status	Married	Married	Divorced	Widowed
Household composition	Caregiver (wife), patient (husband), daughter (temporarily cohabiting during recovery)	Caregiver (daughter), patient (mother), husband	Caregiver (daughter), patient (mother), father	Caregiver (wife), patient (husband: deceased at the time of the interview), daughter (48 years), son (42 years)
Educational level	4th grade	4th grade	12th grade	9th grade
Occupation	Factory worker (garment manufacturing)	Cook (kitchen assistant)	Administrative clerk	Textile industry laboratory technician; florist
Employment status	Retired	Retired	Unemployed	Retired
Duration of follow-up by the CPCST	Approximately 6 months	2 years	2–3 months	Approximately 6 months

Content analysis: interviews

Referral Process Theme

In terms of the Referral Process theme, three families were referred by their family doctor.

“It came through the family doctor.” - C.46

In one case, the referral to the CPCST was made by the hospital while the patient was an inpatient.

“…when she was discharged (…) the doctor said it would be appropriate for her to be referred to the Palliative Care team.” - C.48

With regard to the timeframe between referral and the first visit by the CPCST, only one caregiver provided specific dates. The timeframe was described more vaguely in the remaining cases.

“…when she was discharged on 11th January 2024, the doctor said that it would be appropriate for her to be followed by Palliative Care. (…) The appointment took place in June 2024.” - C.48

“…I can’t say exactly (…) We didn’t wait very long.” - C.46

Caregiving Experience

Care Dynamics: Caregivers described taking on most of the caregiving workload, particularly in relation to hygiene care, feeding, medication management, and accompanying patients to appointments.

“The daily hygiene, I’m the one who does it in the morning.” - C.47

“I feed her; she can already eat some things on her own. But she still needs someone to prepare the meal…” - C.47

“At night I would separate everything into little groups: in the morning this, at lunch that, and then she had the supplements.” - C.49

“I go with them to the appointments…” - C.48

In only one case was there greater support and sharing of tasks among those cohabiting with children.

“My children would… carefully, they would bring him downstairs.” - C.49

As the caregiving period progressed, it was also found that some caregivers needed to hire additional human resources, such as home care services or home-based physiotherapy.

“I do his usual hygiene every day, and they come once a week to wash his hair and to do certain things for him.” - C.47

“The therapist would come (…) so that he wouldn’t lose muscle.” - C.49

Physical/Psychological: Caregivers reported sleep disturbances across all cases.

“Worse, because I wasn’t sleeping…” - C.47

Weight gain resulting from reduced physical activity and changes in eating patterns was also mentioned, as it can impact body image.

“…I gained about 10 kilos, something that really bothers and frustrates me.” - C.48

“I gained more weight. Because it may not seem like it, but I was much more sedentary. And then there was the bread… For me, that was the worst (…) sometimes I would turn to it a bit more.” - C.49

Caregivers also expressed feelings of “duty,” “obligation,” “dedication,” and “love,” which were counterbalanced by “tiredness.”

“…I can’t complain because it is my duty, isn’t it? It’s my duty. But I do have hard days.” - C.46

“It was a choice, a necessity, an obligation to be here, to care.” - C.48

“I am here out of love and dedication, and I will continue to be. But… yes, yes, it doesn’t stop being complicated, it doesn’t stop being wearying…” - C.48

Participants also mention feelings of helplessness or an inability to improve the care provided.

“…because we always want to do more and do better, and we can’t, and we don’t know how.” - C.48

“No matter how much we try to cheer them up, the reality of end of life, the sadness, their loneliness… it affects the caregiver too, because the caregiver, no matter how much they want to, can’t always find the words to encourage them…” - C.48

Witnessing the progression of a disease was described as “difficult” and distressing.

“It is very, very hard to see them deteriorate; it’s horrible.” - C.49

Social Life: Taking on the role of carer led to a significant and progressive reduction in social activities, which, in some cases, ceased entirely. Even when opportunities for social interaction remained, these moments were accompanied by feelings of anguish and anxiety related to leaving the patient alone or in the care of others. As a result, caregivers often choose to restrict their social life, contributing to a growing concentration of all care responsibilities on themselves.

“I used to have one day a week when I went out with some friends. (…) I stopped that a long time ago…” - C.46

“I can’t go on holidays, I can’t go away for a weekend, can I? I can’t, because my brother stays here, but it’s not the same. And it’s not because of my brother. With my brother, she would be fine. It’s because of my mother. She is so used to having me.” - C.48

“I could leave them, but my concern doesn’t let me. I felt anxious, stressed (…) The pleasure of the moment isn’t worth it.” - C.48

Family: Within the Family category, and consistent with previous findings of this study, caregivers reported assuming most caregiving tasks. However, there were situations in which they needed to involve other family members, which affected the family’s daily dynamics.

“Either my daughter takes me (…) and my husband stays here. She can never stay alone. Either my daughter-in-law has to stay, or my son.” - C.47

This was particularly evident in a family where the children lived in the household. The carer described the effect this had on her children’s work and social lives, and she also expressed concern about their mental health.

“He would sometimes miss work, or arrive late; my son was always going in outside his scheduled hours…” - C.49

“My children didn’t get married-unfortunately for them, they had to take care…” - C.49

“My son fell into a very deep depression.” - C.49

As the caregivers were family members of the patients, some also described the impact on their relationships with the people receiving care, including their marital relationships.

“Even though I get very annoyed with him, it’s not his fault.” - C.46

Another caregiver further identified her caregiving situation as a factor that contributed to her divorce.

“I got divorced-it will be a year now this month. Also, very much because of this situation.” - C.48

Professional/Financial: It was observed that, in two cases, taking on the role of carer did not necessarily lead to unemployment, but prevented those individuals from returning to work.

“…I was weighing whether to find another job or care for my mother…” - C.48

In one case, the carer expressed concern about becoming financially insecure upon retirement.

“I have 36 years of contributions, I am 54 years old. (…) I’m too young to retire, too old to find another job. (…) And I need to complete my pension, because if I stay here for however many years taking care of my parents, I won’t be able to make enough contributions.” - C.48

From a financial perspective, the caregivers reported specific expenses relating to the care of the patient.

“We had to buy a chair ourselves.” - C.47

“…no, and the home care assistants come here because we pay for them.” - C.47

In one case, the carer explained that expenses need to be shared among household members.

“When I buy the supplements, I pay. If it’s my son who buys them, he pays. If it’s my daughter, I pay her back.” - C.49

Social Responses and Support: Caregivers mainly referred to financial benefits, such as the disability certificate and the subsidy for purchasing diapers. Some expressed dissatisfaction at the discontinuation of the latter.

“…he had that benefit related to the disability certificate…” - C.49

“He had some kind of subsidy for diapers (…) And then suddenly it was taken away.” - C.49

Expectations

The content included in the Expectations theme was limited and entirely absent from the Social Life, Family, and Professional/Financial categories. Differences in caregivers’ expectations appeared to be related to their varying levels of prior knowledge about PC.

Care Dynamics: The descriptions ranged from an absence of expectations, as seen in a case where the carer appeared to have little prior knowledge about PC:

“No, I didn’t have any expectations. I was really at zero; I didn’t even know what it was. And so I thought: Let them come, and then I’ll see.” - C.46

On the other hand, some caregivers expected technical or clinical support:

“I wanted to be informed. To know (…) what should be done. So I wanted someone from the field…” - C.49

Physical/Psychological: In cases where some prior knowledge existed, it was often incomplete and influenced by the preconceived idea that PC is only for patients expected to die in the near future. This caused discomfort for caregivers.

“I felt that for her to be referred to Palliative Care, it meant she was very unwell. And that she would only be there for a short time.” - C.47

Social Responses and Support: One caregiver mentioned the expectation that the team’s social worker would assess the quality of care being provided.

“I thought she should come to check whether she was being well cared for…” - C.47

Caregiving Experience-Impact of PC

Within the theme of Caregiving Experience-Impact of PC, most of the content fell within the Care Dynamics, Physical/Psychological, and Social Responses and Support categories. No content was identified for the Social Life, Family, or Professional/Financial categories.

Care Dynamics: The caregivers emphasized the importance of receiving support at home and of the team’s regular follow-up phone calls.

“…they come here or they call. It’s not always necessary for them to come, but the fact that they call to ask how things are, and I already know what they need to know-I have everything written down, I take notes. So by now we understand each other well, and it’s a good support.” - C.48

The caregivers also described other interventions carried out by the team, such as managing medication and coordinating with the home-based rehabilitation team.

“…every now and then they would adjust her medication.” - C.47

“Tomorrow a rehabilitation team is coming.” - C.46

A positive impact was also attributed to the caregiver education provided by the nursing team.

“The nursing staff came to teach us and explain better how to divide the meals, how to measure the liquids…” - C.49

Physical/Psychological: Caregivers described the positive impact of community PC. They reported feeling more supported and safer when providing care for their relatives. This sense of security was closely linked to the team’s accessibility through follow-up phone calls.

“I feel calmer, yes, of course. I know that if I need anything, I can call them and they will answer and help me with whatever is needed.” - C.46

Caregivers also reported feeling more clinically supported.

“…during those six months between my mother’s discharge and the first visit, I felt somewhat alone. Not that I couldn’t meet her needs here at home, but the medical needs, the medical support, the medical monitoring-that was what I was missing.” - C.48

They also emphasized the importance of having their caregiving practices recognized by the team.

“And it was important for them to come here and say, ‘you’re doing it well, this is how it should be.’” - C.49

Social Responses and Support: Caregivers primarily referred to material and logistical support.

“They come every morning to provide hygiene care.” - C.46

“…the team also arranged the chair for me…” - C.46

They also mentioned that the team’s social worker would provide guidance on completing forms to apply for social support benefits.

“…it was the social worker who took the initiative to bring me the paperwork.” - C.47

Expectations vs. Reality

Within the Expectations vs. Reality theme, caregivers described how their initial assumptions had changed. These included the belief that PC was only for patients who were terminally ill and that it could not be provided at home.

“It’s not only for people who are terminal, not at all. Palliative care isn’t just for that.” - C.46

“Yes, I had already heard about palliative care. I just thought they didn’t come to the house. I thought I had to go…” - C.47

One caregiver noted the lack of support in hiring home-based services, which could have reduced her workload and given her some time to herself.

“I thought, ‘if there’s some kind of support, maybe I can hire someone to come in the afternoons once in a while, and if I want to go here or there, she can stay here.’ But no, they said there was no support, so it has always been me.” - C.47

From the caregivers’ perspective, the organization of resources by the CPCST was perceived as limited regarding human resources.

“They are too few for the amount of work they have, I suppose. Although I don’t feel that they come here in a rush or that they are trying to get rid of us, no.” - C.48

Initially, one family felt that a referral to PC was unnecessary. However, after becoming familiar with the work of the CPCST, they believed that earlier involvement would have been beneficial. The waiting time for admission was also highlighted.

“They didn’t come right away because they had no capacity at the time (…) It’s a pity it wasn’t a little earlier, maybe. Because they would have supported me more, and I would have had fewer scares, I don’t know.” - C.49

Suggestions for Improvement

Regarding the theme Suggestions for Improvement, most suggestions were general, broadly referring to the need to support people at the end of their lives, particularly for families who choose to provide care at home.

“…between considering placing older people in an institution or having them treated at home, and accompanying families and helping them (…) I think it would be good, and it would be a worthwhile investment for the future…” - C.48

Within the context of family support, caregivers also expressed the need for case-by-case assessments. One caregiver suggested that individual counseling would help caregivers navigate the process and to ensure that the support provided is adapted to their specific needs.

“…the possibility of looking at each case individually. ‘Is the patient meant to stay at home with the family? What is it that they want? We have these options, this one and this one’-which one fits their needs best?” - C.48

Another suggestion was that professionals working in geriatrics should be given greater recognition and appreciation.

“…people who work in geriatrics (…) if it were up to me, those people… they would get an extra day off and earn more.” - C.48

One caregiver, who was mainly concerned about her future retirement, suggested the possibility of continuing to make pension contributions to social security services while providing care.

“I am only asking the government to give me the chance to make contributions so I can have my pension. Because, from what I understood, legally there is no way. Even informal caregivers (…) The bureaucracy and the back-and-forth were so much around a single issue that no one could understand anything…” - C.48

## Discussion

A central finding of this study is that caregivers have very limited free time. This has a negative impact on several areas of their lives and also limits the scope of the study itself. It was challenging to get caregivers to participate, and sometimes, they had to stop the interview to attend to the patient. This context favored immediate operational responses with less depth when questions required reflection or abstract thinking. Social desirability bias was also identified, whereby caregivers attempted to “please” the interviewer, which may have introduced bias into the responses [16]. Some confusion was also observed regarding the impact of PC on both the caregiving experience and the patient’s clinical condition. In some cases, particularly at the beginning of interviews, caregivers showed some resistance to focusing the discussion on themselves rather than on the person receiving care. This further limited the content that could be analyzed.

Within the themes of Expectations and Caregiving Experience-Impact of PC, no codifiable content emerged in the Social Life, Family, or Professional/Financial categories. This absence may reflect methodological limitations, such as limited time, interruptions and lower comfort with abstraction, and/or limitations in the scope and resources of PC interventions. These interventions tend to focus on clinical safety and symptom management, with less coverage of social, family, and work-related domains. The agreement between the findings from Expectations vs. Reality and Suggestions for Improvement, where the need for predominantly social and governmental support emerges, reinforces this interpretation.

The Expectations vs. Reality findings reveal a perception of insufficient human resources, which aligns with late referrals and long waiting times between referral and the first assessment. This reduces the possibility of early intervention. This perception is consistent with the conclusions of qualitative research conducted with community-based healthcare professionals in Portugal [17] and corresponds with well-documented associations in studies on access and equity in PC [18,19].

Best practice recommendations for PC emphasize the importance of early intervention to ensure effective outcomes. Both national and international literature link late referrals and waiting times to limitations in service capacity and shortages in the PC workforce, a systemic constraint that goes beyond the scope of individual teams [7,20], with caregivers’ perceptions aligning with this. Suggestions for Improvement partly emerged in response to these unmet needs. Caregivers proposed strengthening support for families who choose to care for relatives at home at the end of their lives, as they consider this to be financially advantageous for the state compared with institutional or hospital-based care. The literature supports this consideration of the cost-effectiveness of home-based end-of-life care. Furthermore, caregivers felt that the quality of care provided at home cannot be matched elsewhere [21,22].

The social life dimension appears to be one of the most affected. The lack of time for oneself resulting from the concentration of most caregiving tasks on a single person makes caregivers indispensable. This perception is reinforced by findings from other studies [23,24]. At the same time, this seems to be one of the areas where intervention is most limited. One caregiver reported expecting to be able to hire home-based support so that she could have an afternoon to herself, but this was not possible.

Another caregiver, who suggested in the Suggestions for Improvement that she could make social security contributions to secure her future retirement, was in fact already eligible to do so through the voluntary social insurance scheme integrated in the ECI. However, when asked about this, she said that the bureaucratic demands of the ECI process, particularly for someone with very limited time, were a strong deterrent to pursuing it. The same carer suggested providing families with personalized counseling to help them identify and access the most appropriate support for their particular situation.

The problems identified by caregivers and the proposed solutions cannot be addressed solely through the intervention of a CPCST. Broader social and governmental responses are needed.

In terms of the characteristics of the study sample, it is important to note that, although this is a qualitative study with a small convenience sample, all participants were women. This aligns with international evidence showing that the majority of informal caregivers are women, reflecting an unequal distribution of care responsibilities at a structural level. A lack of formal support, such as services, leave policies, and financial allowances, places a considerable burden on families, particularly women, leading to disadvantages in terms of employment, income and social protection, and further widening gender inequalities [25].

In Portugal, the 2024 revisions to the ECI may address some of the identified issues. Alongside efforts to reduce bureaucratic barriers, the reforms also strengthened the available support measures, including the creation of a pool of substitute caregivers to ensure the primary caregiver’s right to rest [10]. Similar initiatives aimed at recognizing and supporting informal carers have emerged in other Western countries [11,25].

At the European and international levels, Directive (EU) 2019/1158 sets out minimum standards for carers’ leave and workplace flexibility. Several countries have introduced additional measures to strengthen these standards further [26]. In the United Kingdom, the “Unpaid Carer’s Leave” legislation entitles individuals to one week of leave per year, which can be taken in parts and includes employment protection [27]. Additionally, the “Carer’s Allowance” scheme provides weekly payments to individuals who provide at least 35 hours of care per week to someone with a qualifying disability. In France, the “Allocation Journalière du Proche Aidant,” a daily allowance that compensates for some income loss during leave for caregiving, has been increased and extended [28]. In Spain, the “Convenio Especial” enables informal caregivers to make pension contributions under the National Dependency Law [29]. Together, these instruments share the goal of protecting caregivers by safeguarding their time, through leave and respite mechanisms, and protecting their income and career progression through allowances and social security contributions. This aligns with the expectations and suggestions identified in this study.

This study has both merits and limitations. This study’s qualitative design enabled an in-depth exploration of caregivers’ lived experiences and subjective perceptions of community PC. The type of knowledge generated could not have been achieved through any other study design. Data were collected through face-to-face home interviews, which facilitated a contextualized understanding of the realities of caregiving. In addition, focusing specifically on caregivers supported by a community PC team provides original and clinically relevant insights into an underexplored setting, allowing for reflection on the adequacy of current support and on potential directions for improving the quality of care.

However, this study has limitations that should be acknowledged. All participants were women and were recruited from a single community PC team in northern Portugal, which may restrict the diversity of perspectives captured. As such, other relevant themes might have been missed from caregivers with different characteristics. The quality of the interviews was constrained by caregivers’ frequent interruptions to attend to the patients during interviews, which induced operational responses and might have reduced the depth of reflection. A tendency to redirect the focus toward the patient rather than the caregiver may also have influenced the narratives. This was clear, especially at the beginning of the interviews. Despite these limitations, this study provides valuable insight into caregivers’ experiences within community PC and highlights areas of unmet need that warrant further investigation.

## Conclusions

This study highlights the substantial impact that home-based caregiving has on the daily lives of caregivers, particularly the scarcity of personal time and the physical and emotional strain associated with prolonged caregiving. Although community PC provides valuable support, including home visits, technical guidance, and easy communication, several unmet needs remain primarily in the social and financial areas. Improving caregivers’ experiences and safeguarding their well-being requires strengthening early PC involvement and expanding formal social support mechanisms. Integrating community PC more broadly with social policies is crucial to ensuring sustainable and equitable home-based end-of-life care.

## Appendices

Appendix A: Sociodemographic Data Questionnaire

Age:

Gender:

Relationship to the patient:

Marital status:

Household composition:

Educational level:

Occupation:

Employment status:

Follow-up duration by the Community Palliative Care Support Team:

Appendix B: Interview Guide

Referral Process and beginning of follow-up by the Community Palliative Care Support Team (CPCST)

Can you tell me how the CPCST came into contact with you and your relative? (This is to help us understand the referral process, including whether the referral was made by a carer, another professional, or a healthcare unit, and how long you had to wait before follow-up began.)

Had you or your relative previously had any contact with palliative care outside of a community setting (e.g., hospital-based palliative care teams)?

Caregiving Experience (before palliative care)

What is it like to be a carer for a family member at home?

Does anyone else provide care for your family member?

How are the caregiving tasks distributed?

How do you organize the care for your relative? Who is responsible for managing the care? (This includes whether other caregivers are contracted, such as home care services.)

What does being a carer mean to you?

Has anything in your life changed since you took on this role?

What changed?

          How do you feel about these changes? How do you feel about being a carer?

          What impact has being a carer had on your personal life? (moving house, family dynamics, social life)

          What has it meant for your professional and financial life? (unemployment, changes in occupation)

          What impact has it had on your health? (medication, illnesses, mental health)

          What impact has it had on your habits? (sleep, diet, physical activity, and hobbies)

          Have any new needs emerged in your life? Which ones? How have you tried to address these needs, and what support have you found? (Psychological and social support)

          What does this change mean to you?

Expectations regarding palliative care follow-up

What were your expectations (thoughts, doubts, uncertainties, feelings, fears)? How did you expect palliative care to influence the difficulties you mentioned earlier?

Caregiving Experience with palliative care (Impact of palliative care on being a caregiver-focusing on the caregiver and not only on the patient’s comfort)

How did you initially feel, and how did you feel over time?

Did these feelings change? Did you notice any difference in how being a carer affected you?

What differences did you notice? (Explore topics addressed in the previous sections.)

How do you feel now?

Expectations vs. Reality

Did the experience live up to your expectations?

What was different, and why?

What kind of support did you need but did not receive?

How could this support have been provided?

Final message/Suggestions for Improvement

If you had the opportunity to speak with someone “important,” someone with the power to make changes, what would you say? (An open space for reflection)
